# Editorial: Indoor environmental quality in day-care centers, kindergartens, and primary schools

**DOI:** 10.3389/fped.2026.2005928

**Published:** 2026-10-01

**Authors:** Dadi Zhang, Hailin Zheng

**Affiliations:** 1 Department of Building Environment and Energy Engineering, The Hong Kong Polytechnic University, Hong Kong, China; 2 Department of Built Environment, Eindhoven University of Technology, Eindhoven, Netherlands

**Keywords:** children, classroom, daycare, indoor air quality (IAQ), indoor environmental quality (IEQ)

Children spend a substantial part of their daily lives in day-care centers, kindergartens, and primary schools. The quality of these environments has shown significant impacts on children's health, comfort, learning, and development (1). Children may be especially susceptible to environmental hazards because of their developing physiological systems, activity patterns, frequent contact with floors and shared surfaces, and higher ventilation rates relative to body size (2). However, the buildings serving young children are remarkably diverse, ranging from nurseries, day-care centers, kindergartens, and primary schools. Their environmental conditions are shaped not only by design, but also by maintenance, occupancy, staff practices, outdoor pollution, and resource constraints.

This Research Topic was established to promote multidisciplinary discussion of indoor environmental quality (IEQ), encompassing indoor air quality (IAQ), thermal comfort, lighting, acoustics, and their interactions (3). Although visual and acoustic quality remain important research priorities, the focus of the articles collected in this Research Topic reflects the urgency and maturity of current evidence concerning IAQ, including ventilation, particulate matter, filtration, hygiene, and contaminated surfaces. These articles also demonstrate that healthier educational environments cannot be achieved through technology alone: effective interventions require appropriate operation, staff engagement, verification, and sustained institutional support.

Díaz-López et al. provided a systematic review of recent *in situ* evidence from day-care centers, preschools, kindergartens, and primary schools. Their study distinguished early childhood settings from primary classrooms. Specifically, in day-care centers, staff-mediated routines, cleaning activities, floor-level contact, and conservative thermal management were especially influential, while in primary schools, IAQ was more strongly affected by longer teaching blocks and schedule-related CO₂ accumulation. The review identified occupancy, ventilation operation, seasonality, outdoor pollution, and moisture conditions as interconnected determinants of exposure. Importantly, it cautioned against treating CO₂ as a universal indicator of air quality. Although CO₂ is valuable for assessing ventilation performance, it cannot independently characterize particulate matter, nitrogen dioxide, volatile organic compounds, or moisture-related hazards. The review translated this evidence into a conceptual three-tier pathway. Initial measures include monitoring, operational routines, and source control; intermediate measures add visible feedback, defined response triggers, filtration, and accountability; and advanced measures address persistent building-level limitations through controlled ventilation, commissioning, and moisture or envelope remediation. This framework reinforced that performance should be evaluated during occupied periods and under seasonally challenging conditions rather than inferred from daily or building-wide averages.

Xia et al. evaluated enhanced filtration in diverse child-serving facilities in Detroit. Their findings showed that portable air cleaners and upgraded heating, ventilation, and air-conditioning filters can substantially reduce particulate matter in suitable settings. However, effectiveness varied across building types. Standard classrooms and a nursery achieved clearer improvements than portable buildings, large multipurpose spaces, or residences converted into childcare facilities. Outdoor pollution, occupancy, cooking, resuspended dust, building leakage, system operation, and room geometry all affected outcomes. This study illustrated why an air cleaner cannot be considered independently of the building and its users. Filters must be appropriately sized, installed, maintained, and operated for sufficient periods. Staff concerns about noise, drafts, energy use, or disruption could reduce actual performance. Therefore, training and clear operational responsibility were not ancillary components but integral parts of filtration interventions. The work also highlighted an environmental-justice issue: facilities in highly polluted communities may have the greatest need for protection while facing older infrastructure and limited maintenance resources.

The importance of human practice is further highlighted by Ishola et al., who examined infection-prevention knowledge and behavior among day-care caregivers in Ogbomoso, Nigeria. Their survey identified substantial gaps in knowledge and implementation, including practices related to hand hygiene, cleaning, waste management, separation of sick children, and shared items. Knowledge and professional experience were associated with infection-prevention practices. While the cross-sectional and self-reported design limits causal interpretation, the study demonstrated that environmental health in childcare settings depends on everyday routines as much as on physical infrastructure. Regular training, supervision, adequate supplies, and enforceable standards are essential, particularly in settings where resources and formal oversight may be limited.

Seong and Hoque extend the discussion from airborne exposure and caregiver behavior to microbial interactions with indoor materials. Their laboratory study showed that bacterial attachment and detachment depend on contact time, surface properties, and near-surface airflow. Rough or porous materials such as carpet and wood created heterogeneous microenvironments that promote retention while also permitting resuspension under certain conditions. Smooth, non-porous surfaces offered more predictable behavior and may facilitate cleaning and disinfection. These mechanistic findings complemented field-based studies by showing that material selection, airflow, cleaning, and occupant activity jointly influence microbial pathways. Nevertheless, because the experiments used a single bacterial strain under controlled conditions, further validation is needed before these findings can be applied to real educational settings.

These contributions support a systems perspective on IEQ. Ventilation can reduce occupant-generated pollutants while increasing the ingress of outdoor particles. Filtration can lower particulate exposure but does not correct inadequate ventilation or control all gaseous pollutants. Cleaning can reduce microbial reservoirs but may generate particles or volatile emissions if products and schedules are poorly selected. Surface materials affect both cleanability and resuspension, while staff behavior determines whether intended protections are sustained.

Future research should expand integrated assessment of thermal, acoustic, and visual quality, while examining how these domains interact with air quality and infection prevention. Healthy learning environments are not defined by a single sensor, threshold, or technology, but by coordinated and equitable systems that place children's health and development at the center of building design and operation.
